# Targeted Therapy in Hepatobiliary Pancreatic Cancer (HPC): Advantages and Advancements of Antibody Drug Conjugates, a Type of Chemo-Biologic Hybrid Drugs

**DOI:** 10.3390/ijms27062707

**Published:** 2026-03-16

**Authors:** Tushar Deb Nath, Attrayo Mukherjee, Subhash C. Chauhan, Debasish Bandyopadhyay

**Affiliations:** 1 School of Integrative Biological and Chemical Sciences (SIBCS), University of Texas Rio Grande Valley, Edinburg, TX 78539, USA; tushardeb.nath01@utrgv.edu; 2 School of Biotechnology, Kalinga Institute of Industrial Technology (KIIT), Patia, Bhubaneswar 751024, India; attrayomukh@gmail.com; 3 Division of Cancer Immunology and Microbiology, Medicine, and Oncology Integrated Service Unit, School of Medicine, The University of Texas Rio Grande Valley, McAllen, TX 78504, USA; subhash.chauhan@utrgv.edu; 4 South Texas Center of Excellence in Cancer Research (ST-CECR), McAllen, TX 78504, USA; 5 School of Earth, Environmental, and Marine Sciences (SEEMS), University of Texas Rio Grande Valley, Edinburg, TX 78539, USA

**Keywords:** hepatobiliary pancreatic cancer, hepatocellular carcinoma, antibody-drug conjugate, drug-antibody ratio, chemo-biologic drug, cytotoxic payload, solid tumors, targeted therapy, pancreatic cancer

## Abstract

Currently, there are very few efficient treatment options for hepatobiliary pancreatic cancer (HPC), which comprises pancreatic ductal adenocarcinoma (PDAC), biliary tract cancer (BTC), and hepatocellular carcinoma (HCC). The HPC tumors are the most lethal malignant tumors in the world. Traditional chemotherapy offers little survival benefit and is associated with notable systemic toxicity, which has made antibody–drug conjugates (ADCs) a hopeful treatment option. Strong cytotoxic drugs combine with monoclonal antibodies to attack tumor-associated antigens. This review discusses the benefits and current developments of Antibody–Drug Conjugates (ADCs) in treating HPC. It also covers their mechanisms of action, ongoing clinical trials, and the challenges of targeting specific antigens like B7-H3, c-MET, and Trop-2. ADCs deliver chemotherapy directly to cancer cells while protecting healthy tissues. It also addresses the favorable outcomes of several preclinical and clinical studies and highlights future paths to enhance ADC efficacy, including addressing tumor heterogeneity, overcoming resistance, and optimizing drug-delivery techniques. This approach has the possibility to further increase patient survival and minimize side effects in HPC patients. To the best of our knowledge and based on the available literature, we have made every effort to include all relevant publications; any inadvertent omissions are entirely unintentional.

## 1. Introduction

Hepatocellular carcinoma (HCC), biliary tract cancer (BTC), and pancreatic ductal adenocarcinoma (PDAC) are among the most aggressive and deadly cancers in the world [1]. Together, hepatobiliary and pancreatic cancers are responsible for approximately 788,000 and 360,000 deaths from cancer each year, respectively, and make up a significant percentage of cancer-related deaths [2,3]. Since hepatobiliary pancreatic (HBP) cancer rates are still increasing, they are playing a bigger role in cancer-related deaths. Despite this alarming trend, there are few treatment options available, especially for those diagnosed at advanced stages, and they typically offer only modest survival benefits. The late-stage diagnosis, bad prognosis, and absence of efficient early diagnosis biomarkers are particularly concerning for PDAC, which accounts for over 90% of pancreatic cancer cases. PDAC is therefore one of the deadliest cancers in the world, with a five-year survival rate of only 12% [4]. Traditionally, chemotherapy has been the mainstay for treating HBP cancers; its efficacy, however, is sometimes constrained and severe off-target effects result from the nonspecific character of cytotoxic drugs [5]. Standard treatment regimens, such as gemcitabine with nab-paclitaxel and FOLFIRINOX for PDAC, along with sorafenib for HCC, have shown only modest increases in median overall survival and progression-free survival. For example, the FOLFIRINOX regimen achieves a median overall survival (mOS) of 11.1 months, with a moderate objective response rate (ORR) of 31.6% [6]. For PDAC patients, the combination of gemcitabine and nab-paclitaxel results in a median overall survival (mOS) of only 8.7 months [7]. These findings enhance efficacy and reduce toxicity, emphasizing the pressing need for creative treatment approaches. The development of antibody–drug conjugates (ADCs) is among the most hopeful breakthroughs in cancer therapy. This targeted therapy, which combines the potent cytotoxic effects of linked drugs with the specificity of monoclonal antibodies (mAbs), provides a more accurate and efficient method of directly treating cancer cells [8]. The “magic bullet” approach targets tumor-associated antigens (TAAs) on cancer cells, selectively preserving healthy tissue and reducing the systemic toxicity commonly seen with traditional chemotherapy [9]. ADCs have the potential to greatly widen the therapeutic window compared to traditional chemotherapies by directly targeting and delivering highly toxic cytotoxic agents to tumor sites. The field has rapidly progressed since the FDA approved Mylotarg^®^, the first ADC, in 2000. Currently, 14 ADCs are approved for the treatment of different types of cancer [10]. ADCs are gaining popularity as a potential innovation in the context of HPB cancers because they can target specific antigens overexpressed in cancer cells, such as HER2 in breast cancer and B7-H3 in HCC. In preclinical and early clinical trials for pancreatic cancer, adjuvants such as sacituzumab govitecan (IMMU-132) have demonstrated significant antitumor activity [11]. The clinical landscape is rapidly evolving with promising initial findings, and a number of ADCs are undergoing trials for the treatment of HPC cancer [12]. Issues like tumor heterogeneity, an immunosuppressive tumor microenvironment, and challenges in achieving accurate antigen targeting continue to limit the broader use of ADCs in HPC [13]. This paper highlights the design, target antigens, and encouraging findings from preclinical research and ongoing clinical studies of ADCs in HPC malignancies. The development of ADCs as a targeted therapeutic approach for these aggressive and treatment-resistant tumors will also be covered, along with significant challenges and future directions. By providing a thorough analysis of ADCs in HPB cancers, this paper seeks to demonstrate the potential influence of these innovative therapies to alter treatment paradigms and enhance survival outcomes for patients with HCC, BTC, and PDAC.

## 2. General Mechanism of Action of ADCs

To specifically eradicate cancer cells while minimizing damage to healthy tissue, monoclonal antibodies are coupled with cytotoxic agents [14,15,16]. These constructs, known as antibody–drug conjugates (ADCs), integrate the specificity of antibodies with the high potency of small-molecule drugs. The monoclonal antibody component targets a specific antigen, typically a tumor-associated protein highly expressed on malignant cells but not in normal tissues, consequently boosting therapeutic selectivity. By binding to the overexpressed antigen on tumor cells, the antibody enables target-specific delivery of the therapeutic agent [17,18,19]. Antigen density, accessibility, and internalization rate all have a significant impact on ADC efficacy, so choosing the right antigen is crucial.

Receptor-mediated endocytosis internalizes the ADC-antigen complex and delivers it to the lysosome, where the cytotoxic payload is released via enzymatic or chemical degradation of the linker, which may or may not be cleavable [20,21,22]. In particular, peptide-based cleavable linkers often require lysosomal enzymes such as cathepsins for linker cleavage. The released drug disrupts microtubules, induces DNA damage, or impairs cellular replication, ultimately resulting in cancer cell death [20,23,24]. Only a few drug molecules are needed to produce cytotoxic effects due to the high potency of ADC payloads.

Some ADCs exhibit a bystander effect, meaning they can help treat heterogeneous tumors by allowing the released drug to spread to nearby antigen-negative cells [25,26,27]. This phenomenon is particularly significant in solid tumors, characterized by frequent heterogeneous antigen expression. For newer non-internalizing ADCs, internalization is not always necessary. These ADCs are designed to overcome difficulties such as low antigen internalization or inefficient endocytic trafficking by exploiting tumor-specific extracellular conditions, such as acidic pH or specific enzymes, to trigger drug release. This strategy permits the drug to be absorbed by surrounding tumor tissue [27,28,29], consequently extending the therapeutic effect of ADCs beyond antigen-positive cells.

Recent advances have addressed obstacles such as low internalization, drug resistance, and tumor heterogeneity by improving target engagement, optimizing payload delivery, and circumventing resistance mechanisms. Notable advancements encompass bispecific ADCs, which target two distinct antigens, and dual-payload ADCs, which deliver multiple cytotoxic agents simultaneously [30,31,32]. Bispecific targeting enhances tumor selectivity, while dual-payload strategies simultaneously disrupt multiple cellular pathways.

ADCs function through a modular and adaptable mechanism, allowing each component of the antibody, linker, and payload to be independently optimized to boost therapeutic efficacy. This technique balances critical factors by guaranteeing precise drug delivery, controlled release at the target site, and potent cytotoxicity. Collectively, these constituents maximize the clinical impact of ADCs [10,33,34].

## 3. Antibody–Drug Conjugates in Hepatobiliary Pancreatic Cancer

**Figure 1:**
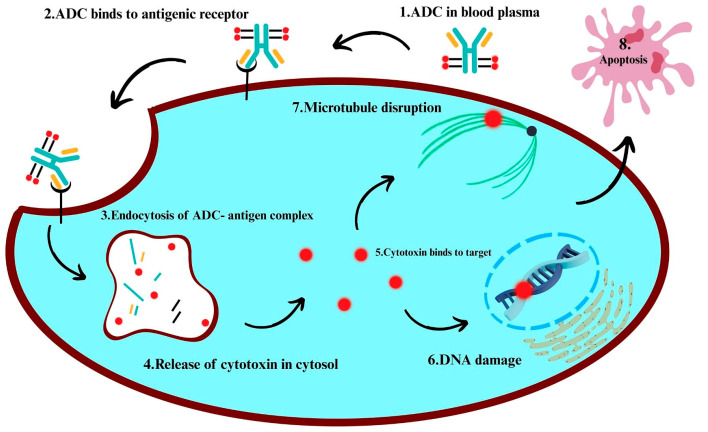
Mechanism of action of antibody drug conjugates.

**Figure 2:**
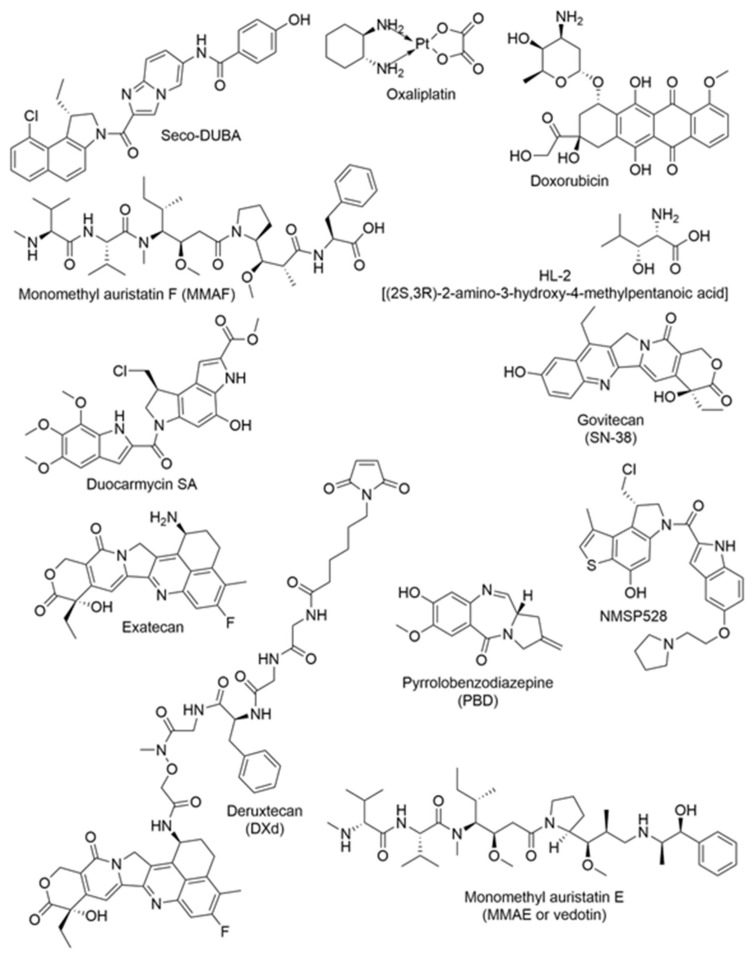
Chemical structures of representative payload drugs employed in the development of Anti-Hepatobiliary Pancreatic Cancer ADCs (Table 1).

An ADC is a targeted therapy formed by covalently linking a monoclonal antibody to a potent small-molecule cytotoxic drug, typically via a linker. This process generally produces a tri-partite structure consisting of an antibody, linker, and payload, although some ADCs are bi-partite when the antibody is directly conjugated to the payload (Figure 1). Most clinically utilized monoclonal antibodies are immunoglobulin G (IgG) molecules, which possess a Y-shaped structure composed of two identical heavy and two identical light chains. This configuration facilitates antigen recognition and immune effector functions. The antibody component enables selective binding to tumor-associated antigens, while the linker must remain stable in circulation and degrade in a controlled manner after internalization to release the cytotoxic payload within tumor cells [35]. Adopting a target-centered approach (Table 1), this narrative review provides an overview of ADC medicines that have demonstrated promising efficacy against Hepatobiliary Pancreatic Cancer (HPC) tumors [36]. The component antibody and payload utilized to make a specific ADC, their therapeutic targets, and clinical trial stages are summarized in Table 1. Figure 2 illustrates the chemical structures of the drugs used as payloads in the ADCs.

### 3.1. ADC Targeting Membrane Antigens in Hepatobiliary Pancreatic Cancer

Targeting Antibody–drug conjugates (ADCs) target membrane antigens by using proteins that are overexpressed on the surfaces of cancer cells to release their payloads inside the cells, enter the cells through receptor-mediated endocytosis, and bind to specific receptors [27,37]. Membrane targets are attractive in hepatobiliary pancreatic (HPB) cancers, including hepatocellular carcinoma (HCC), biliary tract cancer (BTC), and pancreatic ductal adenocarcinoma (PDAC), due to their frequent overexpression [10,38]. This “classic” ADC strategy maximizes direct cytotoxicity to tumor cells while protecting normal tissues with low antigen expression [39]. However, as discussed in Section 3.2 on stroma-targeted approaches, dense stromal barriers in these tumors, particularly in PDAC, may limit ADC penetration and efficacy [39,40].

#### 3.1.1. Anti-B7-H3 Antibody–Drug Conjugates

B7-H3, a type I transmembrane protein with 316 amino acids, belongs to the B7 protein family (sharing 20–27% sequence similarity) and exists as two isoforms, 2Ig and 4Ig [41]. It is found both inside and outside cells, including the nucleus, cytoplasm, cell membrane, and as a soluble form [42].

B7-H3’s role in Hepatocellular Carcinoma (HCC) is significant, serving as both a prognosis marker and a therapeutic target. Though minimally expressed in healthy tissue, it is highly prevalent in HCC tumors (80–93.8%) [43]. Circulating B7-H3 (also called CD276) in serum is a potential tool for identifying high-risk cirrhotic patients and diagnosing HCC earlier than standard biomarkers [44]. By enhancing cell proliferation, adhesion, migration, and invasion, B7-H3 promotes HCC progression. Through mechanisms involving MMPs and EMT, it suppresses the activity of CD8+ T cells [45,46]. Poor patient outcomes and more advanced stages of the illness are linked to higher levels of B7-H3. As a result, including vobramitamab duocarmazine (Table 1), clinical trials are currently investigating therapies targeting B7-H3 [47].

In pancreatic adenocarcinoma (PAC), B7-H3 is frequently elevated with positivity rates between 66% and 93.2%, and higher expression is linked to more aggressive tumors [48,49,50]. Through the TLR4/NF-κB pathway, B7-H3 promotes PAC spread and reduced survival rates after surgery, as reported in [51]. B7-H3 also contributes to chemotherapy resistance, but silencing it can restore treatment sensitivity [49]. Targeting B7-H3 with antibodies or cellular therapies, such as CAR-T cells, has demonstrated promising anti-tumor effects, including enhanced immune cell infiltration and tumor reduction, as shown in vivo [52]. Clinical trials are also investigating agents such as vobramitamab duocarmazine for the treatment of PAC [53].

In addition to vobramitamab duocarmazine, several other B7-H3-targeting antibody–drug conjugates (ADCs) are undergoing clinical trials for advanced solid tumors, specifically including hepatobiliary pancreatic cancers (HPC) such as pancreatic ductal adenocarcinoma (PDAC), hepatocellular carcinoma (HCC), and biliary tract cancer (BTC). Ifinatamab deruxtecan (I-DXd; DS-7300a), a potential first-in-class B7-H3-directed DXd ADC (humanized anti-B7-H3 IgG1 monoclonal antibody linked to a topoisomerase I inhibitor DXd via a cleavable peptide linker), has demonstrated encouraging antitumor efficacy in preclinical models and initial trials [54]. It showed an objective response rate (ORR) of up to 48–55% in phase 2 studies of extensive-stage small cell lung cancer (e.g., IDeate-Lung01, NCT05280470), which led to FDA Breakthrough Therapy Designation in 2025 [55]. The phase 1b/2 IDeate-PanTumor 02 trial (NCT06330064) specifically includes cohorts for PDAC, HCC, and BTC, among other solid tumors, evaluating efficacy and safety in pretreated patients with recurrent or metastatic disease; enrollment is ongoing with a focus on 12 mg/kg Q3W dosing (8 mg/kg starting for HCC safety run-in) [56]. Ifinatamab deruxtecan is safe enough to use, with common grade ≥ 3 events like neutropenia and anemia, and low rates of interstitial lung disease [55]. MediLink Therapeutics developed YL201, a new B7-H3-targeted ADC that uses a tumor microenvironment-activatable linker-payload platform linked to a topoisomerase I inhibitor (YL0010014; DAR ~8) [57]. In a large global phase 1/1b trial (NCT06057922 and related studies) enrolling heavily pretreated patients with advanced solid tumors (including pancreatic cancer, *n* = 10), YL201 achieved an overall ORR of ~41% and disease control rate of ~84%, with early signals of partial responses in pancreatic and other tumors; no strong correlation was observed between B7-H3 expression and response [57]. Safety was satisfactory, characterized predominantly by neutropenia, leukopenia, and anemia (grades ≥ 3 in 25–32%), with minimal interstitial lung disease (~1%) [57]. Phase 3 trials are progressing in various indications, indicating potential for HPC expansion [57]. Finally, 7MW3711 (Mabwell), a new B7-H3 ADC on the IDDC™ platform (interchain-disulfide drug conjugate with a topoisomerase I inhibitor payload), has shown promising efficacy in phase 1/2 studies (e.g., NCT06008379) for advanced solid tumors, including partial/complete responses in esophageal and lung cancers (ORR 39–50% at ≥4.0 mg/kg doses); while HPC-specific data are emerging, the broad solid tumor enrollment and combination trials with PD-1 inhibitors suggest relevance for B7-H3-overexpressing HPC [58,59]. These new B7-H3 ADCs demonstrate strong potential for treating HPC, particularly in addressing heterogeneity and resistance. They also support more focused research into these aggressive cancers [59].

#### 3.1.2. Anti-c-MET Antibody–Drug Conjugates

c-MET, a receptor tyrosine kinase, is frequently overexpressed in hepatobiliary and pancreatic cancers. Antibody–drug conjugates (ADCs) targeting c-MET are currently under investigation as potential therapies [36,60,61]. c-MET promotes aggressive tumor behaviors, including invasion, metastasis, angiogenesis, and epithelial–mesenchymal transition (EMT), all of which contribute to poor prognosis in solid tumors. Dysregulation of c-MET signaling may result from overexpression, gene amplification, ligand-dependent activation, or pathway crosstalk with other oncogenic drivers, establishing c-MET as a clinically relevant biomarker and therapeutic target. Activation by hepatocyte growth factor (HGF) stimulates cell growth and survival [60,61,62,63,64,65]. In hepatobiliary and pancreatic (HPB) malignancies, c-MET activity is also linked to resistance to targeted therapies and chemotherapy, underscoring the need for strategies that directly eliminate c-MET–high tumor cells rather than solely inhibiting signaling. Intratumoral heterogeneity in c-MET expression can influence treatment response, highlighting the importance of sampling strategies and assay thresholds for patient stratification in clinical trials. ADCs deliver cytotoxic agents directly to tumor cells, thereby reducing systemic side effects. Certain c-MET ADCs may also induce a “bystander effect,” in which membrane-permeable payloads impact adjacent antigen-low cells. This effect may be advantageous in heterogeneous tumors such as pancreatic ductal adenocarcinoma (PDAC) and biliary tract cancer (BTC) but must be balanced against the risk of off-tumor toxicity, as c-MET is expressed at low levels in some normal tissues. In PDAC, a highly lethal cancer, c-MET is frequently overexpressed [60,65]. The dense stromal microenvironment in PDAC can impede the penetration and distribution of large antibody-based therapeutics, making factors such as linker chemistry, drug–antibody ratio (DAR), and antibody affinity critical for effective delivery and efficacy. Combination strategies that modify the tumor microenvironment or enhance immune infiltration are being evaluated alongside c-MET–directed ADCs. Novel c-MET ADCs, including SHR-A1403 (Table 1), have demonstrated significant anti-tumor activity in preclinical models by inhibiting cancer cell growth and migration and inducing cell death through disruption of the cell cycle and metabolism. In some instances, c-MET ADCs such as ABBV-399 are being tested in combination with immunotherapies [60,66], with promising results observed in hepatocellular carcinoma (HCC). Combining c-MET ADCs with immune checkpoint inhibitors may enhance antigen presentation and inflammatory signaling, potentially increasing tumor responsiveness to immunotherapy. Careful monitoring for overlapping toxicities is essential, particularly when combining cytotoxic payloads with immunotherapies. Dual-targeting ADCs that address both c-MET and RON are being developed to overcome resistance, as these designs may reduce signaling redundancy and compensatory pathway activation, which often lead to relapse after single-target therapies. Next-generation ADC formats, such as biparatopic antibodies or conditionally activated ADCs, are being explored to improve selectivity and minimize systemic toxicity. Similar strategies may benefit c-MET–directed platforms. Key challenges include selecting optimal drug payloads, preventing resistance, and optimizing antibody specificity. Additional considerations involve ensuring linker stability in circulation to prevent premature payload release, while enabling efficient intracellular drug release after endocytosis. Resistance mechanisms may include reduced internalization, altered lysosomal processing, increased efflux transporter activity, or antigen downregulation, each of which necessitates tailored ADC engineering or combination approaches. Accurate patient selection based on c-MET expression is essential for effective treatment. Standardized companion diagnostics, such as immunohistochemistry (IHC) scoring systems or genomic amplification assays, and clear expression cutoffs are required to identify likely responders and interpret clinical trial outcomes. Research into c-MET ADCs for these cancers is ongoing [63].

#### 3.1.3. Anti-CD147 Antibody–Drug Conjugates

CD147 is a transmembrane glycoprotein that plays a pivotal role in cancer progression by promoting cell proliferation, invasion, and metastasis through its interactions with cancer-associated fibroblasts and induction of matrix metalloproteinases (MMPs). Mechanistically, CD147 forms signaling complexes, such as those with integrins and MCT1/MCT4, which activate the MAPK/ERK, PI3K/AKT, and NF-κB pathways. This activation results in increased expression and secretion of MMPs, particularly MMP-2 and MMP-9, as well as other pro-invasive factors. Furthermore, CD147 stimulates adjacent fibroblasts to acquire a cancer-associated fibroblast (CAF) phenotype, leading to the release of additional MMPs and cytokines that enhance extracellular matrix remodeling, invasion, and metastasis [66]. Antibody–drug conjugates (ADCs) targeting CD147 are under investigation as therapeutic strategies for aggressive hepatobiliary and pancreatic cancers, which currently lack effective treatments. CD147, also known as EMMPRIN, is overexpressed in these malignancies and is integral to angiogenesis, metastasis, and tumor development by regulating matrix metalloproteinases and vascular endothelial growth factors. ADCs consist of an antibody directed against CD147 conjugated to a cytotoxic agent, facilitating selective tumor cell destruction, inhibition of tumor growth and metastasis, and modification of the tumor microenvironment [67].

Several clinical trials are evaluating anti-CD147 ADCs like Mehozumab-DM1, HAP18, and Metuximab. However, challenges include potential toxicity to normal tissues expressing CD147, drug resistance, and the need for improved ADC design and patient selection [67].

It is highly expressed in hepatocellular carcinoma (HCC) tumor cells, with low or absent expression in healthy tissue. A meta-analysis of 880 HCC patients found a significant correlation between CD147 expression and TNM stage, as well as with relapse-free survival (RFS) and disease-free survival (DFS) [68].

One promising ADC, Anti-CD147-ILs-DOX (Table 1), combines the anti-CD147 antibody metuximab with pegylated liposomal doxorubicin (Doxil) [69]. In vitro, it was efficiently internalized by CD147-positive HCC cells, with IC50 values ranging from 9.64 to 58.24 μg/mL. In vivo, it showed significantly higher potency than free doxorubicin, Doxil, or Doxil combined with metuximab, suggesting it may provide a more effective targeted treatment for HCC [36].

#### 3.1.4. Anti-HER3 Antibody–Drug Conjugates

Recent studies underscore the significant role of HER3 as both a prognostic indicator and a therapeutic target in pancreatic cancer [70]. A study examining HER family receptor expression (HER1, HER2, HER3, and HER4) in operable pancreatic cancer found that HER3 overexpression was associated with a poorer prognosis. Patients with HER3 expression had a notably shorter median survival (12 months) than those without it (25.6 months). Conversely, patients who were HER4-positive had a more favorable survival outcome, suggesting that HER3 could be a reliable independent prognostic marker for pancreatic cancer [71,72].

Beyond its prognostic significance, HER3 has emerged as a therapeutic target, particularly in combination with radiation [73]. A new HER3-targeting antibody–drug conjugate (ADC) has been demonstrated to enhance the radiation response in pancreatic ductal adenocarcinoma (PDAC) models. This combination led to enhanced tumor regression and better survival rates, highlighting the potential of HER3-targeting ADCs as effective radiosensitizers in cancer treatment [68,74].

In subsequent studies, the EV20/NMS-P945 ADC (Table 1), designed to target HER3 across various solid tumors, has shown promising results. This ADC, which pairs the anti-HER3 antibody EV20 with the potent cytotoxic agent NMS-P528, demonstrated excellent therapeutic efficacy and stability in preclinical models, including pancreatic cancer. With its favorable pharmacokinetics and potent cytotoxic effects, EV20/NMS-P945 holds promise as a new treatment option for HER3-positive cancers [75,76].

Another promising development is AMT-562 (Table 1), an ADC that links exatecan, a topoisomerase I inhibitor, to an anti-HER3 antibody. AMT-562 has demonstrated superior efficacy in preclinical models of pancreatic, colorectal, and lung cancers, especially in tumors with low HER3 expression. With potent antitumor activity and a favorable safety profile, AMT-562 offers a potential approach to overcome resistance to existing HER3-targeted therapies. These findings reinforce the importance of HER3 as a therapeutic target and suggest that ADCs targeting HER3 could serve as an effective treatment for HER3-expressing cancers, including pancreatic cancer [70,77].

#### 3.1.5. Anti-CD133 Antibody–Drug Conjugates

The role of CD133, also known as prominin-1, in cancer, particularly in hepatocellular carcinoma (HCC), has been widely studied due to its involvement in tumorigenesis, recurrence, resistance, and metastasis to treatment. CD133 is a transmembrane glycoprotein that acts as a marker for cancer stem cells (CSCs) in numerous tumors, including liver, pancreas, and colorectal cancers [78]. CSCs are a small subset of cancer cells that can proliferate and differentiate, properties crucial for tumor initiation and growth [79]. CD133+ cells, representing this subpopulation, are frequently linked to a poor prognosis for cancer patients because of their role in driving tumor growth and promoting metastatic spread [80].

In HCC, CD133+ cells have been shown to contribute significantly to chemotherapy resistance, particularly to drugs such as 5-fluorouracil (5-FU) and vincristine. This resistance is partly attributed to the activation of key survival pathways, including the Akt/PKB signaling pathway and the NOTCH pathway [78,81]. Studies have indicated that inhibiting these pathways can sensitize CD133+ cells to chemotherapy, offering potential strategies for enhancing treatment efficacy. Specifically, pre-incubating HCC cells with inhibitors of the NOTCH pathway, such as DAPT, has been shown to decrease the population of drug-resistant CD133+ cells and increase apoptosis in response to chemotherapy [81]. This highlights the critical role that these signaling pathways play in maintaining CSCs’ resilience to standard cancer therapies.

Moreover, high expression of CD133 in HCC tumors correlates with advanced disease stages, higher tumor grades, and shorter survival times. Patients with elevated CD133 expression tend to experience higher recurrence rates after treatment, further underscoring the relevance of CD133 in cancer progression. This correlation between CD133 and poor prognosis suggests that targeting CD133+ cells could be an effective strategy to reduce tumor relapse and metastasis, both of which are major challenges in cancer treatment [80].

Additionally, CD133+ cells have been shown to adapt to the tumor microenvironment, which often includes hypoxic conditions and nutrient deprivation. In these environments, CD133 expression is upregulated, aiding CSC survival by promoting increased glucose uptake and autophagy [82]. These metabolic adaptations help CD133+ cells survive in hostile conditions, contributing to their aggressiveness and ability to form tumors even in nutrient-poor environments [83]. This metabolic reprogramming is another key feature that makes CD133+ CSCs particularly difficult to target with traditional therapies.

#### 3.1.6. Anti-CD24 Antibody–Drug Conjugates

Antibody–drug conjugates (ADCs) targeting CD24 are emerging as a promising strategy for treating hepatocellular carcinoma (HCC), a malignancy often associated with poor prognosis and high recurrence rates [84]. CD24, a mucin-like protein, is overexpressed in HCC tissues and correlates with tumor invasiveness, metastasis, and activation of critical signaling pathways such as Wnt/β-catenin [84,85]. Studies show that high CD24 expression in HCC is linked to a more aggressive phenotype and poor patient outcomes, making it a potential biomarker for predicting recurrence after surgery [86,87,88].

Several ADCs targeting CD24 are under investigation, including G7mAb-DOX (Table 1), which combines a monoclonal antibody targeting CD24 with doxorubicin, a potent chemotherapeutic agent [83,89]. This ADC has shown significant in vitro and in vivo efficacy, selectively targeting CD24-positive tumor cells, inhibiting tumor growth, and prolonging survival in HCC mouse models [83,89]. Notably, a critical advantage over traditional chemotherapy, this ADC reduces systemic toxicity by delivering the cytotoxic drug directly to tumor cells [83,90]. Moreover, CD24-targeted therapies may be especially beneficial for HCC patients with normal α-fetoprotein (AFP) levels, who traditionally face challenges in monitoring tumor recurrence. However, despite the promise of ADCs, challenges such as potential off-target effects and the development of drug resistance remain [16,62,91,92]. Future research will focus on optimizing ADC designs, enhancing specificity, and identifying biomarkers to predict treatment responses [93,94].

#### 3.1.7. Anti-Glypican-3 Antibody–Drug Conjugates

In cancers like hepatocellular carcinoma (HCC), cholangiocarcinoma (CCA), and pancreatic ductal adenocarcinoma (PDAC), Glypican-3 (GPC3) protein is often found at high levels. GPC3 is a part of the glypican family. This glypican family is made up of six heparan sulfate proteoglycans. It is under investigation as a target for antibody–drug conjugates (ADCs). Specifically, these ADCs use an antibody that binds to GPC3 on cancer cells and delivers a potent cell-killing drug directly to the tumor. Once these ADCs bind to the GPC3 protein on the cancer cell, they are internalized, and the drug is released, causing the cancer cell to die.

In 63.6% to 72% of hepatocellular carcinoma cases, GPC3 expression was observed, while healthy liver tissues tested positive for GPC3 in only 9.2% [95,96]. This protein is involved in several key signaling pathways, such as HGF/c-MET, HIPPO/YAP/TAZ, and Wnt/β-catenin, which promote tumor progression, HCC cell invasion, and migration [97]. The presence of GPC3 in malignancies has been linked to a lower five-year overall survival (OS); patients with GPC3-positive tumors had an OS of 54.5%, compared to 87.7% for those with GPC3-negative tumors [98].

Using a dipeptide linker, a humanized IgG1 anti-GPC3 monoclonal antibody is linked to a pyrrolobenzodiazepine (PBD) dimer (HYP7-PC) or duocarmycin SA (HYP7-DC) (Table 1). Two GPC3-targeted antibody–drug conjugates (ADCs) are developed [99]. The drug-to-antibody ratios (DAR) were 1.7 and 2.4 for HYP7-DC and HYP7-PC, respectively [5]. PBDs, a new type of drug payload, damage DNA by crosslinking it, and they offer advantages like rapid breakdown, good cell penetration, and a ‘bystander effect’ that kills nearby cells, all while limiting overall drug exposure [100]. With successful trafficking to the lysosomes, the resulting ADCs showed effective but slow internalization. In vitro, respectively, with IC50 values of 11 ng/mL and 50 ng/mL, the HYP7-DC and HYP7-PC demonstrated activity against GPC3-positive HCC cell lines. In reducing the growth of tumors and extending OS in vivo, HYP7-PC was more significant compared to HYP7-DC, which only slowed the growth of the tumor in HCC xenograft mouse models. Additionally, the antitumor effect is increased by the combination of HYP7-DC with gemcitabine. With no significant changes in body weight, both ADCs were well tolerated [36].

#### 3.1.8. Anti-Trop-2 Antibody–Drug Conjugates

In various human carcinomas, including ovarian [101], colorectal [102], pancreatic [103], gastric [104], and squamous cell carcinoma of the oral cavity [105], a type-I transmembrane glycoprotein Trop2 is widely overexpressed [106,107]. It is initially identified on human trophoblast cells. A critical role in cellular calcium signaling [108] and oncogenic pathways, such as the activation of ERK [109], is played by Trop2. Its appeal as a therapeutic target stems from its elevated expression in poor patient prognosis. With studies indicating it is present in most patient tumors, specifically between 94% [110] and 99.5% [103], ductal adenocarcinoma (PDAC) shows a very high prevalence of Trop2 expression. Its significance as a therapeutic target is underscored by the presence of Trop2 in advanced PDAC and its association with reduced patient survival [110].

FDA-approved ADC Sacituzumab govitecan (SG, TRODELVY^®^) (Table 1) is used for metastatic triple-negative breast cancer, which is under investigation for pancreatic adenocarcinoma (PDAC) [111]. SG consists of a humanized anti-Trop-2 IgG1 monoclonal antibody linked to the topoisomerase I inhibitor SN-38 and induces DNA damage, ADCC, and apoptosis [111,112]. With a 4.5-month median overall survival in 16 PDAC patients in a Phase I/II trial, it produced no objective responses, though SG showed efficacy in other cancers [113]. Alongside capecitabine for PDAC and with radioimmunotherapy to enhance efficacy while minimizing toxicity, as shown in the SG study, even though prior results have been limited [114].

The potent topoisomerase I inhibitor is linked to a humanized anti-Trop-2 IgG1 monoclonal antibody via the ADC Dato-DXd (Datopatomab deruxtecan). DXd is 10 times more powerful than the active metabolite of irinotecan, SN-38 [115,116]. Its clinical evaluation in the TROPION-PanTumor01 Phase I trial is driven by the significant antiproliferative activity of Dato-DXd, as shown in PDAC Preclinical studies. Early results from the cohort with non-small cell lung cancer showed encouraging antitumor effects, with a 26% objective response rate and a median overall survival of 11.4 months, though some patients had grade ≥ 3 treatment-related adverse effects and drug-related lung interstitial disease [117]. Bystander killing was achieved using the same linker and payload; a control ADC also showed substantial tumor growth inhibition [113]. Sharkey et al. explored that in preclinical cancer models, pancreatic cancer ADCs combine with radioimmunotherapy (RIT) [118]. In these studies, the hRS7-SN-38 ADC showed some antitumor activity, with a cure rate of only 10%. When combined with Yttrium-90-labelled anti-mucin PAM4 antibody, cure rates increased to 10% with single doses of 2.8 MBq and 50% with a 4.8 MBq single dose. The combination of hRS7-SN-38 and 90Y-hPAM4 achieved higher cure rates of 40% and 90% at the same doses, and complete cures were achieved with a combined plan that included a chemotherapeutic approach (gemcitabine), RIT, and ADC. Modification of the humanized anti-Trop2 antibody RN927C, with precise linker and drug-payload conjugation, showed a dose-dependent reduction in tumor growth, outperforming conventional gemcitabine chemotherapy in pancreatic and patient-derived xenograft models [119].

#### 3.1.9. Anti-EGFR Antibody–Drug Conjugates

Epidermal growth factor receptor (EGFR) is a key transmembrane glycoprotein that plays a crucial role in cell proliferation, angiogenesis, metastasis, and the regulation of apoptosis [120]. It is primarily expressed in epidermal and epithelial cells and in the pancreas, where it is upregulated in 30 to 90% of pancreatic cancer cases, driving pro-survival and anti-apoptotic pathways [121]. Given its role in cancer progression, EGFR is a prime goal for cancer treatments, including antibody–drug conjugates (ADCs). However, many patients develop resistance to anti-EGFR therapies, which has spurred the development of novel EGFR-directed ADCs to improve treatment efficacy.

Low-affinity humanized EGFR antibodies were developed to selectively target cancer cells with high EGFR expression while minimizing toxicity to normal cells. In vitro studies showed that low-affinity antibodies bound effectively to high-EGFR-expressing cancer cells, whereas uptake by normal epidermal cells was reduced. The low-affinity EGFR ADC (RN765C) demonstrated potent cytotoxicity against moderate- to high-EGFR-expressing cancer cell lines and minimal damage to normal epidermal keratinocytes, demonstrating efficacy in a pancreatic xenograft model. This strategy showed promise in reducing side effects while improving therapeutic efficacy [122].

A humanized anti-EGFR monoclonal antibody (RC68) was conjugated to Monomethyl auristatin E (MMAE) using two cleavable VC linkers. The new PY linker improved serum stability for both ADCs. Both ADCs exhibited comparable binding affinity and antitumor activity in a pancreatic cancer xenograft system, outperforming gemcitabine treatment. Similarly, an EGFR ADC (Epidermal Growth Factor Receptor Antibody–Drug Conjugate) using a dibromopyridazinedione linker to improve serum stability and uniform drug-to-antibody ratio was developed. This ADC effectively overcame resistance in EGFR mAbs associated with KRAS mutations, a common feature in pancreatic cancers [122].

Further research by Greene et al. involved Cetuximab, an EGFR-directed ADC linked to MMAE (Table 1). Cetuximab disrupted EGFR-ligand interactions, inducing receptor internalization and degradation [121,123]. In vitro, this ADC demonstrated superior potency against pancreatic cancer cell lines with varying EGFR expression, and it showed significant survival benefits in xenograft models compared with cetuximab or placebo alone [123]. Similarly, Li et al. generated ADCs using a humanized anti-EGFR mAb (RC68) conjugated to MMAE with either mc-vc-PAB or py-vc-PAB linkers [124]. Both ADCs showed strong internalization in EGFR-positive cells and limited tumor growth in xenograft models, demonstrating their potential for treating pancreatic cancer.

LR-DM1 (Table 1), an ADC composed of LR004 (a chimeric anti-EGFR mAb) conjugated to DM1, showed potent antiproliferative activity in vitro and displayed a broad therapeutic index in vivo [120]. The treatment was effective at doses far below the lethal dose, highlighting its potential as a treatment for pancreatic cancer. Furthermore, RN765C (Table 1), a low-affinity anti-EGFR ADC, showed selective uptake by cancer cells with high EGFR expression while sparing normal tissues, effectively inducing tumor regression in multiple solid tumor xenografts [125].

#### 3.1.10. Anti-Glypican-1 Antibody–Drug Conjugates

Glypican-1 (GPC1) is a heparan sulfate proteoglycan that is attached to the plasma membrane through glycosylphosphatidylinositol. While GPC1 is either absent or minimally present in healthy pancreatic tissue, it is significantly overexpressed in pancreatic cancer, and this overexpression is linked to poorer survival outcomes [126]. Research by Tsujii and colleagues demonstrated that most pancreatic cancer patients exhibit GPC1 expression in both tumor and stromal cells, particularly in cancer-associated fibroblasts. Based on this, they developed an antibody–drug conjugate (ADC) targeting human GPC1, using MMAF (Monomethyl auristatin F) or MMAE (Monomethyl auristatin E) linked via a VC linker. Both ADCs effectively killed GPC1-positive pancreatic cells in laboratory models and in patient-derived xenografts (PDX) [126,127,128]. In a model with GPC1 expression in the stroma but not the tumor, the MMAE ADC performed better than the MMAF conjugate. This was attributed to the ADC being processed by CAFs, which released MMAE, which then killed surrounding cells, including bystander tumor cells [127].

GPC1 has been controversial as a pancreatic cancer biomarker because it occurs in two forms: a membrane-bound protein and a soluble fragment shed from the membrane. Circulating exosomes from PDAC patients show high GPC1 levels that correlate with disease severity, although similar exosomes from patients with benign pancreatic diseases also express GPC1 [129]. This raises questions about the reliability of GPC1 as a diagnostic biomarker [130].

GPC1 is a promising target for targeted therapies, particularly in pancreatic cancer, where it is overexpressed in up to 80% of cases [131,132]. GPC1 is found in both tumor and stromal cells, making it an ideal target for stroma-targeting ADC development. In contrast, normal pancreatic cells show little to no GPC1 expression. GPC1 overexpression is significantly associated with more advanced disease stages and worse survival rates [125,126,132]. Nishigaki and colleagues designed a GPC1-directed ADC using a mouse-derived anti-human GPC1 antibody conjugated to MMAF via a mc-vc-PABC linker [127]. This ADC demonstrated excellent internalization and effectiveness against PDAC cell lines, with significantly lower IC50 values than unconjugated MMAF. The ADC inhibited tumor cell growth through G2/M cell cycle arrest and apoptosis in PDX models [133].

The researchers later created a humanized version of the GPC1 ADC, conjugating it to MMAE, and observed strong selectivity for GPC1-positive cells, with no effects on GPC1-knockout cells. This ADC also showed potent, dose-dependent cytotoxicity in several GPC1-positive PDAC cell lines. Furthermore, it exhibited a significant bystander effect, effectively killing neighboring tumor cells in heterogeneous GPC1-expressing PDAC models. Importantly, the GPC1-MMAE ADC (Table 1) demonstrated substantial antitumor activity in PDX models that included both GPC1-positive CAFs and GPC1-heterogeneous tumor cells, further supporting its potential in treating pancreatic cancer [125].

#### 3.1.11. Anti-Mesothelin Antibody–Drug Conjugates

An intriguing target for antibody-based cancer treatments is mesothelin, a protein whose expression is modest in healthy tissues but dramatically increased in many malignancies, including carcinomas. It is an attractive therapeutic target since it is expressed in over 90% of pancreatic tumors [134,135,136]. Pancreatic cancer is one of many pre-clinical cancer models that have examined the efficacy of BAY 94-9343, an anti-mesothelin antibody–drug conjugate [137]. The human pancreatic cancer cell line MIA PaCa-2 was found to overexpress mesothelin, making the cells more susceptible to the anti-mesothelin ADC, according to these investigations. Results showed that the ADC had a dosage-dependent effect in a subcutaneous pancreatic cancer model, with three injections of the highest dose producing a full response. When administered at the maximum dose, the ADC surpassed the gold-standard therapy, gemcitabine, and produced transient tumor shrinkage in a patient-derived xenograft (PDX) model (PAXF736) [137].

One such ADC, AMA-MMAE, has shown potential for the management of pancreatic cancer, among other indications. Mesothelin expression was detected in vitro in four pancreatic cancer cell lines; however, there was no association between mesothelin expression levels and ADC response in vivo. While in vivo experiments proved that this was not caused by mesothelin downregulation, PET imaging with 89Zr-anti-mesothelin ADC revealed decreased tumor uptake in the unresponsive mice. This was likely due to lower antigen internalization in these models, resulting in reduced release of the active drug. Mesothelin, a cell-surface glycoprotein anchored by a GPI anchor, shows minimal expression in healthy tissues but is highly upregulated in numerous solid tumors. It promotes PDAC cell proliferation by activating STAT3 and autocrine IL-6 signaling, and also promotes tumor invasion by increasing matrix metalloproteinase activity. In PDAC patient specimens, mesothelin was expressed in 89–91.6% of cases, but its expression did not correlate with clinicopathological data [136].

Anetumab ravtansine (AR) (Table 1) is another ADC targeting mesothelin, composed of a human anti-mesothelin IgG1 antibody connected to the maytansine derivative, DM4 (Table 1), by use of a reducible disulfide linker, with an average drug-to-antibody ratio of 3.2. Preclinical tests showed that AR had potent cytotoxic effects in vitro, with an IC50 of 0.72 nM, and inhibited tumor growth in xenograft systems. AR also induced a significant bystander effect, affecting adjacent mesothelin-negative cells, making tumors with heterogeneous antigen expression [138] effective. In a Phase I clinical trial (NCT01439152), AR was tested in 148 patients with advanced or metastatic solid tumors, including 9 patients with PDAC [139]. Three of the PDAC patients had stable disease. In the cohort receiving the highest tolerated dose, 55% of patients experienced grade ≥ 3 treatment-related adverse events; 8% discontinued treatment, and 47% required a dose reduction. In a subsequent phase II study (NCT03023722) of pretreated advanced PDAC patients, two patients showed stable disease, while 12 had disease progression. Additionally, in the NCI10208 phase Ib trial (NCT03816358), AR was combined with nivolumab, with or without gemcitabine or ipilimumab, for pretreatment mesothelin-augmented advanced PDAC. Of the 25 evaluable patients, no objective response rate (ORR) was observed, although 12 patients had persistent illnesses. Among the eight patients in the AR plus gemcitabine cohort, all had stable disease, and this combination will be further explored in the study’s expansion phase [140,141].

#### 3.1.12. AG-7

AbGn-107 (Table 1) is an antibody–drug conjugate (ADC) that targets the AG-7 antigen, a Lewis A-like glycol-epitope present in 24–61% of gastric, colorectal, pancreatic, and biliary cancers. Because AG-7 is a carbohydrate-based glyco-epitope rather than a conventional protein receptor, its expression is determined by tumor glycosylation patterns and may vary across histological subtypes and disease stages. This variability has significant implications for patient selection and biomarker testing. Such antigens are attractive ADC targets due to their high tumor association, although expression heterogeneity remains a major limitation in solid tumors. AbGn-107 comprises a humanized anti-AG-7 IgG1 monoclonal antibody linked to the tubulin inhibitor DM4 via a proprietary cleavable linker. The cleavable linker and maytansinoid payload, such as DM4, are intended to enable effective intracellular release following endocytosis and lysosomal processing and may also facilitate bystander killing depending on the properties of the payload and linker. Here, it is worth noting that maytansinoids are highly potent mitotic inhibitors that interact with tubulin, leading to G2/M-phase cell-cycle arrest and ultimately triggering cell death. Achieving an optimal balance between linker stability in circulation and release within the tumor microenvironment is critical to minimizing systemic toxicity. AbGn-107 was evaluated in a phase I, first-in-human dose-escalation trial (NCT02908451), which was terminated early due to operational issues. Early-phase dose-escalation studies are primarily designed to define safety, identify dose-limiting toxicities, and recommend a phase II dose; thus, limited antitumor responses are anticipated, particularly in heavily pretreated populations. The early termination of this study limits interpretation of exposure–response relationships and precludes robust conclusions regarding therapeutic benefit in specific tumor cohorts, such as pretreated pancreatic ductal adenocarcinoma (PDAC). The trial enrolled 35 patients, including 20 with heavily pretreated PDAC, across six dose levels. The inclusion of a substantial PDAC cohort is notable given the historically low response rates to systemic therapies in advanced PDAC, highlighting the importance of identifying tumors with sufficient AG-7 expression and ensuring effective drug delivery through the dense pancreatic tumor stroma. Future studies should incorporate prospective biomarker enrichment and standardized immunohistochemical or glyco-epitope detection methods to confirm target positivity. The drug demonstrated a favorable tolerability profile, with grade 3 or higher treatment-related adverse events (TRAEs) occurring in 14.2% of patients. For ADCs with microtubule-inhibiting payloads, clinically relevant adverse events may include hematologic toxicity, neuropathy, fatigue, and ocular effects, depending on payload and linker design. Therefore, reporting the spectrum of adverse events and exposure at each dose level is essential for contextualizing tolerability. The relatively low rate of high-grade TRAEs in this small cohort suggests feasibility, but larger studies are necessary to more confidently define the safety profile. The best-observed response was durable stable disease lasting more than six months in two patients, although it is unclear whether these individuals had PDAC. Durable stable disease in refractory solid tumors can be clinically meaningful, particularly when accompanied by symptom control or biomarker improvement. However, without tumor-type–specific breakdowns and imaging-based response data, interpretation of the signal remains challenging. Stratified reporting by AG-7 expression levels and prior lines of therapy would help determine whether benefit correlates with target abundance or specific clinical contexts. Despite preliminary evidence of effectiveness, no further data have been published on the use of AbGn-107 in pancreatic cancer [142]. This gap highlights a broader challenge in ADC development for hepatopancreatobiliary (HPB) malignancies: early signals may be lost due to operational constraints, funding priorities, or strategic reorientations, even when a target is biologically plausible. Future research should prioritize clearer translational endpoints, including target expression, internalization, pharmacokinetics, and payload-related biomarkers, as well as transparent cohort-level reporting, to strengthen the evidence base for AG-7–directed ADCs in PDAC and biliary cancers.

#### 3.1.13. Claudin 18.2

Claudin 18.2 is an isoform of claudin 18, a transmembrane protein essential for tight junction structure and function [143]. It is expressed in 54.4% to 70% of PDAC patient samples, with strong expression associated with better differentiation and improved survival [144,145,146,147].

SOT102 (SO-N02) (Table 1) is an anti-CLDN18.2 monoclonal antibody conjugated to PNU-159682, a topoisomerase II inhibitor. Preclinically, SOT102 showed favorable pharmacokinetics and achieved a complete response in PDAC patient-derived xenografts, independent of CLDN18.2 expression. However, a phase I/II trial, at the sponsor’s discretion, led to the termination of advanced PDAC [148].

CMG901 (Table 1) is an ADC targeting CLDN18.2 with MMAE as the payload, demonstrating potent antineoplastic activity. A phase I study showed significant efficacy in patients with gastric cancer, with a 100% disease control rate and a 75% objective response rate. 11% had Grade 3 or higher treatment-related adverse effects. PDAC efficacy results are pending [149].

SYSA1801 (Table 1), a fully human anti-CLDN18.2 IgG1 monoclonal antibody conjugated to MMAE, showed superior antineoplastic activity against PDAC in preclinical studies. Early-phase I results showed a 38.1% ORR and a 57.1% DCR for patients with gastric cancer, with 24.2% experiencing grade ≥ 3 adverse events [150].

RC118 (Table 1), an anti-CLDN18.2 monoclonal antibody conjugated to MMAE, is in a phase I/II trial for sophisticated cancers, including PDAC, but no preclinical data have been published. TORL-2-307 (Table 1), another CLDN18.2-directed ADC, is under evaluation in a phase I trial for advanced malignancies, including PDAC [36].

#### 3.1.14. Tissue Factor (TF/CD142)–Targeted Antibody–Drug Conjugates

Tissue factor (TF), or CD142, is a protein that activates blood clotting after tissue injury and is overexpressed in many malignancies, including pancreatic cancer. In tumors, TF promotes growth, angiogenesis, and metastasis, which worsens prognosis. It is found in 54–89% of PDAC patient samples, with strong expression correlating to poor differentiation and higher rates of cancer-associated venous thromboembolism [151,152].

TF targeting several antibody–drug conjugates (ADCs) has been developed. These ADCs showed promising results in preclinical studies, particularly in pancreatic cancer models, where they demonstrated dose-dependent anti-tumor effects. The anti-human TF ADCs were more effective than anti-mouse TF ADCs, with greater tumor uptake and reduced stroma localization. Additionally, ADCs targeting TF in low-expression models still elicited tumor responses, likely due to ADC cleavage and bystander killing in the tumor microenvironment [153].

In vivo testing of a bis-alkylated anti-TF ADC showed improved tumor control at lower doses in murine pancreatic cancer xenografts. This effect was partly due to reduced TF expression and endothelial cell killing [154]. A humanized anti-TF mAb, when administered as an ADC, attenuated tumor growth in subcutaneous pancreatic cancer xenografts [155]. Moreover, SC1, a mouse anti-TF mAb, reduced pancreatic cancer cell migration and tumor growth both in vitro and in vivo [156].

Clinical trials are ongoing with ADCs like XB002, which targets TF with an auristatin payload. Early results from a phase I study show stable disease in some patients with PDAC, with further trials planned to assess efficacy [157].

#### 3.1.15. CEA (CEACAM5): Biological Rationale and Therapeutic Targeting via ADCs

Carcinoembryonic antigen (CEA), also known as CEACAM5 or CD66e, is a cell-surface glycoprotein typically expressed in gastrointestinal tissues during fetal development. However, it is overexpressed in several cancers, including pancreatic cancer [158].

A CEA-targeting antibody drug conjugate is developed by conjugating SN-38 to labetuzumab, a humanized IgG1 monoclonal antibody, using a linker that can be cleaved. In preclinical studies, the labetuzumab-SN-38 ADC (Table 1) significantly improved survival in a xenograft model of human pancreatic cancer, demonstrating superior outcomes compared with therapies using free antibody or free drug [159].

A CEA-targeting mouse monoclonal antibody was used to conjugate to a near-infrared fluorophore and paclitaxel. While the ADC showed reduced effectiveness in vitro compared to free paclitaxel, it successfully targeted and reduced tumor growth in vivo, although statistical significance was not reached [160].

A humanized CEACAM5 monoclonal antibody conjugated to DM4 (SAR408701) via a SPDB linker demonstrated in vitro effectiveness versus pancreatic cancer cell lines, although it was not tested in pancreatic cancer models. The ADC showed effectiveness in treating PDXs of colon, lung, and stomach cancers [161].

Tusamitamab ravtansine (TR), an ADC combining a humanized anti-CEA monoclonal antibody with the maytansinoid derivative DM4, showed a strong antiproliferative effect in vitro against pancreatic cancer cells. In vivo, TR showed strong antineoplastic effects against PDXs of various solid tumors [160]. A phase I trial (NCT02187848) in heavily pretreated solid tumor patients, including PDAC, showed stable disease in 35.7% of patients in the 2-week cohort and 40% in the 3-week group, but no objective responses were confirmed. TR is also being tested in a phase II study (NCT04659603) as monotherapy or in combination with gemcitabine for advanced PDAC [153].

#### 3.1.16. HER2

HER2, a protein that drives cancer growth when overactive, is traditionally targeted in breast and stomach cancers. However, it is also found in biliary tract cancers (BTCs) and pancreatic cancer (PC), making it a potential target for these difficult-to-treat diseases [162]. Antibody–drug conjugates (ADCs), which combine targeted antibodies with potent drugs, are a promising approach that delivers chemotherapy directly to HER2-positive tumor cells [162,163].

BTCs, cancers of the bile ducts and gallbladder, often show HER2 overexpression, particularly in gallbladder cancers. This has led to clinical trials testing HER2-targeting ADCs. Trastuzumab deruxtecan (T-DXd) has shown encouraging results in HER2-positive BTC patients, with significant tumor shrinkage and disease control. Zanidatamab, a bispecific antibody, also shows promise in early trials. Other ADCs, such as RC48-ADC and MRG002, are being further investigated [162].

Pancreatic cancer, a highly aggressive disease, also sometimes shows HER2 overexpression [164,165]. While the frequency varies, HER2-targeted ADCs are being explored as a treatment option. Trastuzumab emtansine (T-DM1), in combination with pertuzumab, has shown potential in pancreatic cancer [153]. Researchers are also exploring the combination of HER2-targeted ADCs with inhibitors of other cancer-driving pathways to overcome drug resistance and improve effectiveness [1].

### 3.2. ADC Targeting the Stroma of Hepatobiliary Pancreatic Cancer

The stroma in Hepatobiliary Pancreatic cancers (HPC), like pancreatic ductal adenocarcinoma (PDAC), and hepatocellular carcinoma (HCC), is dense and made of extracellular matrix (ECM) proteins, CAFs, pericytes, and dysfunctional blood vessels. This results in high fluid pressure and poor permeability, making it difficult for ADCs to penetrate the tumor effectively [39]. In PDAC, the stroma can account for up to 90% of the tumor mass, forming a strong barrier. ADCs designed to target the cell membrane may accumulate in the tumor but fail to penetrate deeply [39].

CAST therapy aims to increase protease activity [39]. Non-internalizing ADCs can release drugs into tumor cells [27]. DAaRTS is another method that enables stromal cells to internalize a prodrug, process it, and then release the free drug, which can then kill surrounding tumor cells [39]. These approaches help enhance the therapeutic effect of ADCs in tumors with dense stroma [27,39,166] and address this by using the ECM as a scaffold, allowing ADCs to bind to and release their payload via such as MMAE proteolytically in the ECM, achieving strong activity without entering

#### 3.2.1. Insoluble Fibrin

Cancer tumors often develop a dense fibrin stroma as a result of tumor-driven blood clotting, creating a unique target for antibody–drug conjugates (ADCs) [167]. Tissue factor (TF), a clotting-promoting molecule abundant in pancreatic and other cancers, triggers this clotting process. ADCs targeting TF, by linking an anti-TF antibody to MMAE, have shown anti-cancer effects in laboratory models of pancreatic cancer. These ADCs accumulate within the tumor, effectively slowing its growth in animal studies. Their large size allows them to penetrate leaky tumor blood vessels while sparing healthy tissues, making them a promising treatment strategy [168].

Additionally, research indicates that combining TF-targeting ADCs with therapies that disrupt the tumor’s supportive stroma could enhance treatment effectiveness by attacking both cancer cells and their blood supply [166]. In a related study, A stroma-targeting ADC is generated by conjugating an anti-insoluble fibrin (IF) IgG mAb to MMAE using a Val-Leu-Lys linker, with an average drug-to-antibody ratio of 3 [168]. This linker is specifically triggered in the presence of IF, which is cleaved by plasmin, a fibrinogen derivative deposited in the tumor microenvironment, making it appropriate for stroma-targeting medications in cancers with abundant stroma [39].

The stability of the generated antibody drug conjugate was confirmed in plasma, and the researchers treated multiple human pancreatic cancer cell lines with IF-MMAE (Table 1), observing strong anti-cancer activity, with IC50 values ranging from 0.16 to 1.19 μg/mL. In vivo, IF-MMAE significantly suppressed tumor growth in PDAC PDX mouse models with abundant tumor stroma, outperforming naked mAb and unconjugated MMAE, excluding major body weight changes [37,39].

#### 3.2.2. ICAM-1

ICAM-1, also called CD54, is a cell-surface protein in the immunoglobulin superfamily that is expressed at high levels on various cancer cells, including pancreatic cancer cells. It is upregulated in cancer cells and contributes to increased invasiveness and metastatic potential. The KRAS-G12D mutation, which is common in pancreatic cancer, drives ICAM-1 expression in acinar cells, thereby attracting macrophages and accelerating the formation of precancerous lesions [169]. ICAM-1 expression is significantly higher in pancreatic cancer cells, with up to 10^6^ molecules per cell, compared to normal pancreatic duct epithelial cells, where ICAM-1 expression is minimal. High ICAM-1 expression correlates with disease stage and survival [170].

Researchers have developed antibody–drug conjugates (ADCs) targeting ICAM-1, carefully designing the antibody, chemical linker, and cell-killing drug to optimize their effectiveness. Laboratory studies show that ICAM-1’s high expression on pancreatic cancer cells, along with its efficient internalization of ADCs, makes it a promising target for treatment. Several ICAM-1 ADCs have been developed, including one with a low-cell-permeable medication and a non-cleavable linker (ICAM1-DM1), which showed the greatest effectiveness against pancreatic cancer cells while sparing normal cells. This ADC significantly reduced tumor growth in a pancreatic cancer orthotopic model by inhibiting tumor cell proliferation and reducing metastatic disease. In animal models, ICAM-1 ADCs have demonstrated significant tumor shrinkage and prevention of recurrence. MRI can be used to noninvasively monitor treatment effectiveness, offering a targeted approach to managing pancreatic cancer [171].

Two ICAM-1-directed ADCs are constructed: ICAM1-DXd using a tetrapeptide spacer and a drug-to-antibody ratio of 8, and ICAM1-MMAE (Table 1), which connects four molecules of the microtubule inhibitor MMAE to the anti-ICAM1 monoclonal antibody. Both ADCs effectively targeted tumors and mediated endocytosis in cancer cells. Although ICAM1-MMAE was more harmful, ICAM1-DXd was less toxic because of the strength of its payload; it showed a stronger bystander-killing effect, targeting more ICAM-1-negative neighboring tumor cells. In vivo, ICAM1-DXd proved more effective than ICAM1-MMAE, partly because it activates anti-tumor immunity by upregulating type I interferon signaling in patient-derived xenograft models [36,153].

#### 3.2.3. Collagen 4

Collagen IV, a key structural protein, is abundant in the tumor microenvironment of liver, pancreas, and bile duct cancers [36]. Researchers are investigating ADCs that target collagen IV as a potential treatment for these cancers. Collagen IV’s role in cancer cell attachment, migration, and survival, coupled with its high concentration in the tumor microenvironment, makes it a promising target for ADC therapy [172]. The approach aims to deliver cytotoxic drugs directly to both the tumor and its supporting tissue, potentially disrupting the tumor’s environment and inhibiting cancer progression [36,173].

To target collagen IV, an antibody–drug conjugate (ADC) is designed, which was intended to target the stroma. IgG monoclonal antibody (mAb) to SN-38 through an ester linker, with a drug-to-antibody ratio ranging from 6.7 to 8.4 [38]. These ester linkers had been specifically chosen for their ability to degrade gradually within the tumor’s extracellular space. The ADC is preferentially attached to the collagen IV matrix, accumulating within the tumor microenvironment. In stroma-rich pancreatic ductal adenocarcinoma (PDAC) patient-derived xenograft models, the ADC was significantly more effective in limiting tumor growth compared to an analogous ADC targeting a tumor cell antigen [36].

Research has shown that high levels of collagen IV are connected to lower survival outcomes in pancreatic cancer, further supporting the rationale for targeting this protein. Additionally, elevated collagen IV levels in the blood after surgery have been identified as a strong prognostic indicator for pancreatic cancer patients [172].

#### 3.2.4. Anti-Fibronectin Extra Domain B (EDB + FN) Antibody–Drug Conjugates

Fibronectin’s extra domain B splice variant (EDB + FN) is a protein that tumor-associated fibroblasts make and that is found in the extracellular matrix (ECM). It is highly expressed in the stroma of various cancers, such as pancreatic ductal adenocarcinoma (PDAC), hepatocellular carcinoma (HCC), non-small cell lung cancer (NSCLC), breast cancer, and ovarian cancer, yet is infrequently found in normal adult tissues [174]. EDB + FN helps tumors grow by helping new blood vessels form, changing the structure of tissues, and making a framework for areas of tumors that do not get enough oxygen.

In Hepatobiliary Pancreatic cancers (HPC), characterized by a stroma-rich environment, constituting up to 90% of the tumor mass, targeting EDB + FN offers a potential avenue for efficient drug delivery [39]. Aur0101 is a human IgG1 antibody linked to the auristatin payload that uses a cleavable linker to target EDB + FN in the ECM. This strategy does not depend on tumor cell internalization; rather, it releases the drug proteolytically in the tumor stroma, resulting in localized drug effects and collateral damage [27,174]. Preclinical studies demonstrate that this ADC exhibits strong efficacy in pancreatic cancer models, resulting in substantial tumor growth inhibition and total tumor regression [174]. When this ADC is used with anti-PD-L1 checkpoint inhibitors, it is more effective by encouraging immune cells to enter the tumor and by reducing tumor size [174].

These encouraging outcomes in preclinical models have led to ongoing clinical trials, including a Phase I study for recurrent/metastatic head and neck cancer, which has demonstrated favorable safety and efficacy. As research advances, EDB + FN-targeted ADCs may surmount the stromal barrier in HPC and enhance outcomes in patients with treatment-resistant disease [39,174].

#### 3.2.5. Anti-Tumor Endothelial Marker 8 (TEM8/ANTXR1) Antibody–Drug Conjugates

TEM8 (also known as ANTXR1) is a transmembrane receptor that is found on cancer-associated fibroblasts, blood vessels, and pericytes. It is most common in tumors with abundant stroma, such as PDAC and HCC. TEM8 is highly expressed in these cancers but is almost absent in normal tissues, making it an ideal target for ADC therapy in HPC. TEM8 promotes tumor growth by remodeling the ECM, forming new blood vessels, and supporting cancer cells in the stroma [174]. The anti-TEM8 ADC m825-MMAE uses a process called Drug Activation and Release Through Stroma (DAaRTS). This method allows stromal cells, such as CAFs, endothelial cells, and pericytes, to take up and store the ADC. The lysosomes process the ADC once it enters, and MMAE is released into the tumor environment, where it kills nearby cells through bystander effects. This mechanism does not require direct binding to tumor cells; instead, it depends on stromal cells to deliver the drug to the tumor [174].

Preclinical studies in pancreatic cancer models demonstrated that this ADC could induce tumor regression, inhibit metastasis, and markedly prolong survival, even when initiated at advanced tumor stages. The ADC was also very effective in models of breast, lung, ovarian, and colon cancer. Animal models showed that it was well-tolerated and did not cause any major side effects. These results indicate that TEM8-targeted ADCs may represent a promising approach for treating stroma-rich tumors, such as Hepatobiliary and Pancreatic cancers, and may enhance therapeutic outcomes when used in conjunction with immunotherapies or other treatments [174].

**Table 1:** An overview of representative ADCs designed for Hepatobiliary Pancreatic Cancer treatment.

Malignancy	Antibody–Drug Conjugates (ADC)	Drug (Payload)	Target	Clinical Trial Phase [Ref]
Hepatocellular carcinoma (HCC)	Vobramitamab duocarmazine	Seco-DUBA	B7-H3	Phase I [38,39,40]
	c-MET-OXA	Oxaliplatin	c-MET	Preclinical [49,50,51]
	SHR-A1403	SHR152852		Phase I [49,50,51]
	ABBV-400	Undisclosed topoisomerase I inhibitor		Phase I [49,50,51]
	Anti-CD147-ILsDOX	Pegylated liposomal doxorubicin	CD147	Preclinical [51,59]
	EV20-sss-vc/MMAF	Monomethyl auristatin F (MMAF)	Human Epidermal Growth Factor Receptor 3 (HER 3)	Preclinical [1,2,3]
	AC133-vcMMAF	Monomethyl auristatin F (MMAF)	CD133	Preclinical [69,70,71,72]
	G7mab-DOX	Doxorubicin	CD24	Preclinical [68,75,84]
	HN-01	HL-2		Preclinical [1,2,3]
	HYP7-DC	Duocarmycin SA	Glypican-3	Preclinical [5,51,62]
	HYP7-PC	Pyrrolobenzodiazepine		Preclinical [5,51,62]
Pancreatic ductal adenocarcinoma (PDAC)	Sacituzumab govitecan	SN-38	Trop-2	Phase I/II [103,104,105]
	Datopatomab deruxtecan (Dato-DXd)	DXd		Phase I [103,107,108,109,110,111]
	AMT-562	Exatecan		Preclinical [61,68]
	EV20/NMSP945	NMSP528		Preclinical [66,67]
	HER3-MMAF	Monomethyl auristatin F (MMAF)		Preclinical [15,92,93]
	Vobramitamab duocarmazine	Seco-DUBA	B7-H3	Phase I [9,10,11]
	GPC1-MMAF	Monomethyl auristatin F (MMAF)	Glypican-1	Preclinical [120,122]
	GPC1-MMAE	Monomethyl auristatin E (MMAE or vedotin)		Preclinical [120]
	Anetumab ravtansine	DM4	Mesothelin	Phase I (NCT03023722, NCT03816358) [126,127,132]
	AbGn-107	DM4	AG-7	Phase Ia (NCT02908451) [134]
	SOT102	PN8-159682	Claudin18.2	Phase I/II [139]
	CMG901	Monomethyl auristatin E (MMAE or vedotin)		Phase I [140]
	SYSA1801	Monomethyl auristatin E (MMAE or vedotin)		Phase I [141]
	RC118	Monomethyl auristatin E (MMAE or vedotin)		Phase I/II [51]
	TORL-2-307	N/A		Phase I [51]
	TF-MMAE	Monomethyl auristatin E (MMAE or vedotin)	Tissue factor	Preclinical [142,144,152,153]
	TF-DM1	Mertansine (DM1)		Preclinical [142,143,153]
	CetuximabMMAE	Monomethyl auristatin E (MMAE or vedotin)	(Epidermal Growth Factor Receptor) EGFR	Preclinical [112,113]
	RC68-mc-vcMMAE	Monomethyl auristatin E (MMAE or vedotin)		Preclinical [112,113]
	LR-DM1	Mertansine (DM1)		Preclinical [118,119]
	RN765c	PF-06380101		Preclinical [114]
	IF-MMAE	Monomethyl auristatin E (MMAE or vedotin)	Insoluble fibrin	Preclinical [51,175]
	Collagen 4-SN-38	SN-38	Collagen 4	Preclinical [51,159,160,161]
Biliary tract cancer (BTC)	Adotrastuzumab emtasine (TDM1, Kadcyla^®^)	Mertansine (DM1)	HER2	Phase II [162]
	Famtrastuzumab deruxtecan (T-DXd, Enhertu^®^)	DXd		Phase II [163]
		Mertansine (DM1)		
	GQ1001	Mertansine (DM1)		Phase I [162]
	Disitamab vedotin (DV)	Monomethyl auristatin E (MMAE or vedotin)	HER2	Phase II [162]
	ICAM1-MMAE	DXd	ICAM1	Preclinical [51,144]
	GPC1-MMAF	Monomethyl auristatin F (MMAF)	Glypican-1	Preclinical [120,122]

## 4. Future Aspects of ADCs in Hepatobiliary Pancreatic Cancer

Recent developments in Antibody–Drug Conjugates (ADCs) have significantly improved the treatment of malignancies, including advanced biliary tract and pancreatic tumors [2,175]. By combining the specificity of monoclonal antibodies with potent chemotherapeutic agents, ADCs enable more precise targeting of cancer cells while reducing harm to healthy tissue. Important ADC improvements include better antibody engineering, maximized linker chemistry, and stronger drug payloads. While developments in cleavable linker technology enable preferential delivery of chemotherapy to the tumor site, thereby reducing side effects, humanized monoclonal antibodies have improved the precision of cancer cell targeting. Furthermore, by influencing neighboring cancer cells, the use of highly potent, membrane-permeable cytotoxins has increased treatment effectiveness [6]. As of January 2025, there are 1768 ADC candidates in development, and among them, 416 are now in clinical trials, most of which are being actively researched [7]. Among the significant ADCs in trials for pancreatic and hepatobiliary tumors are Becotatug vedotin, aimed at EGFR in advanced biliary tract cancer, and Izalontamab brengitecan, targeting both EGFR and HER-3 in advanced biliary tract carcinoma [8,9]. Respectively, for advanced pancreatic and biliary tract tumors, SHR-A1904 and Tecotabart vedotin, both targeting CLDN18.2, are being assessed [10,11]. Trastuzumab deruxtecan, aimed at HER-2, is currently in Phase 3 for advanced biliary tract cancer (NCT06467357) [176]. These ADC innovations show encouraging tailored therapy plans meant to enhance patient outcomes and lower negative consequences in pancreatic and hepatobiliary malignancies [6].

In addition to improving monospecific ADCs, future developments should include bispecific antibody–drug conjugates (BsADCs) that target two antigens at once. This will make tumors more selective, encourage dual-mediated internalization, create stronger bystander effects, and overcome single-antigen heterogeneity, which is especially important for heterogeneous HPC tumors such as PDAC, HCC, and BTC. For example, EGFR/B7-H3 BsADCs (such as IBI-3001 from Innovent Biologics) leverage the co-expression of EGFR (30–90% in PDAC) and B7-H3 (high in HCC and PDAC) to enhance endocytosis and payload delivery while blocking compensatory signaling pathways [35,177]. IBI-3001 has progressed to phase 1 trials (NCT06349408) for advanced solid tumors exhibiting EGFR/B7-H3 co-expression, demonstrating superior preclinical antitumor efficacy and safety compared to monospecific ADCs, with potential relevance to HPC. HER3/MUC1 BsADCs (e.g., the DM002 series) also address co-expression in PDAC. They enable co-internalization and payload switching (for example, to overcome resistance in models like DM002-BLD1102), resulting in strong anti-tumor efficacy in cell-line-derived and patient-derived xenograft PDAC models, outperforming single-target ADCs [178]. Bispecific platforms such as EGFR/HER3 (e.g., BL-B01D1 or iza-bren) have shown promising results in early trials for solid tumors, including BTC-relevant indications [177,179,180]. These BsADCs are an important part of the future plan to make HPC more effective by reducing off-target effects and addressing resistance. This is why ongoing trials need to have dedicated HPC cohorts.

A crucial future objective, particularly for PDAC, is the development of stroma-penetrating or stroma-targeting ADCs to overcome the dense desmoplastic stroma, which constitutes up to 90% of the tumor mass, increases interstitial pressure, hinders vascular perfusion, and significantly limits antibody and drug infiltration [181,182,183]. Strategies include ADCs targeting stromal antigens overexpressed in cancer-associated fibroblasts (CAFs) or in the extracellular matrix, such as glypican-1 (GPC1) on CAFs, that facilitate bystander killing of heterogeneous tumor cells via MMAE/MMAF payloads in PDX models [131]. Another promising approach targets urokinase activator receptor (uPAR), which is found in large amounts in both the tumoral and stromal parts of PDAC. A site-specific uPAR-targeted ADC (for example, FL1-PNU with plasminogena PNU-159682 payload) has been shown to strongly suppress tumors, remodel the stroma, and alter the immune landscape in stroma-dense PDAC models [165]. Complementary innovations encompass smaller-format ADCs, such as nanobody-based conjugates designed to enhance tissue diffusion; stroma-modulating combinations, including ADCs combined with hyaluronidase or FAK inhibitors to diminish ECM stiffness and enhance perfusion; and engineered linkers or payloads that facilitate extracellular release or amplify bystander effects in stroma-rich environments [152]. Validated in preclinical PDX and genetically engineered mouse models, these approaches could synergize with existing HPC-targeted ADCs to overcome penetration barriers, diminish resistance, and enhance outcomes in PDAC [184].

## 5. Conclusions

Antibody–drug conjugates, or ADCs, represent a significant advance in the treatment of hepatobiliary and pancreatic cancers, offering a more precise and potent alternative to conventional chemotherapy. By combining the strong cytotoxic properties of chemotherapy drugs with the targeting ability of monoclonal antibodies, ADCs reduce dangerous systemic exposure and deliver treatment straight to tumors. Despite ongoing challenges with tumor heterogeneity, immunosuppressive tumor environments, and antigen specificity, ADCs have shown encouraging results in clinical trials for HCC, BTC, and PDAC. Further clinical research, as well as developments in linker design, drug payloads, and antibody engineering, are expected to increase their therapeutic potential. Improving these strategies to overcome resistance and enhance targeting could significantly change the paradigm for treating these aggressive tumors [185,186].

## Data Availability

No new data were created or analyzed in this study. Data sharing is not applicable to this article.
